# Repurposing Conventional Magnetic Functional Agents: A Novel Strategy for Long-Acting, Safe, Magnetically Mediated Precision Oncology

**DOI:** 10.3390/pharmaceutics18030319

**Published:** 2026-03-02

**Authors:** Zihan Lv, Yue Wang, Yimin Su, Albert Yu, Rouye He, Zhongjian Xie, Yao Zhu

**Affiliations:** 1 Faculty of Materials Science, Shenzhen MSU-BIT University, Shenzhen 518172, China; 19862210525@163.com (Z.L.); yxz20@tsinghua.org.cn (A.Y.); 2 Shenzhen Children’s Hospital, Shenzhen 518038, China; wangyue650171@163.com (Y.W.); 15859624409@163.com (Y.S.); rouyehe@163.com (R.H.); 3 Affiliated Shenzhen Children’s Hospital of Shantou University Medical College, Shenzhen 518038, China

**Keywords:** magnetic field therapy, magnetic materials, repurposing strategy, glioma therapy, angiogenesis impairing

## Abstract

**Background**: Although conventional magnetic functional agents provide a material basis for magnetically mediated tumor therapy, they have long been restricted by the application framework of magnetic resonance imaging (MRI) or magnetic thermal ablation. **Methods**: This study proposed a repurposing strategy of two mature magnetic functional agents, Fe_3_O_4_ nanoparticles and gadopentetic acid (GA), by applying their unique magnetic response properties to enhance magnetic field therapy for glioma. **Results**: Both magnetic materials, when combined with an external magnetic field (MF), showed a synergistic effect to amplify the therapeutic effect. In the CT-2A glioma-bearing mice model, it resulted in marked suppression of tumor growth, with the growth inhibition (TGI) rate increasing from ~27% after MF therapy alone to 64% and 55% after the Fe_3_O_4_- and GA-potentiated MF therapy, respectively. It was proposed that the MF effect on impairing tumor angiogenesis was enhanced, as evidenced by significant reductions in CD31 expression and microvessel density. It disrupted nutrient supply to the tumor, augmenting the tumor suppression efficiency. The reduced infiltration of CD4+ and CD8+ T cells into tumors further confirmed the effective blockade of tumor perfusion. **Conclusions**: This study established a new paradigm of conventional magnetic materials to enhance the non-thermal physical effects and biological regulatory effects of magnetic field for glioma therapy, instead of only as the imaging agents or magnetic hyperthermia agents.

## 1. Introduction

Magnetic field (MF) penetrates human tissue without side effects to normal cells and shows advantages over invasive treatments such as surgery and radiotherapy. Its therapeutic effects can be precisely controlled by adjusting parameters such as strength, frequency, and exposure duration [1]. Clinically, MF therapy has been applied in diverse areas, including peripheral nerve regeneration [2,3], osteonecrosis [4,5], pain relief [6,7], muscle function regulation [8], anti-inflammatory effects [9,10], and wound healing promotion [11,12]. However, there is no consensus on its mechanisms in tumor treatment. A critical limitation for MF therapy is that MF intensity diminishes with tissue penetration depth, resulting in insufficient strength at target sites for deep-seated tumors. Magnetic materials are emerging as a promising strategy to address this challenge through the synergy of multiple mechanisms such as magnetic targeting, magnetic hyperthermia, magnetic mechanical force, and catalytic reactions.

Notably, magnetic materials can enhance local magnetic field strength at tumor sites and boost the therapeutic performance of MF-based interventions [13]. Among the most promising candidates are superparamagnetic Fe_3_O_4_ nanoparticles (NPs) and paramagnetic gadopentetic acid (GA) agents. Fe_3_O_4_ is an FDA-approved magnetic nanomaterial, with excellent magnetic responsiveness and surface modifiability, enabling interactions with an external MF for functions such as localized heat generation or mechanical actuation [14,15,16,17]. It has been shown to induce significant tumor cell death [18,19]. GA is a clinically used MRI contrast agent, containing Gd^3+^ ions with unpaired electrons that can enhance local magnetic responsiveness [20,21].

In this study, the conventional magnetic functional agents, including Fe_3_O_4_ NPs and GA agents, were repurposed for a novel strategy for long-acting, safe, magnetically mediated precision oncology to enhance glioma therapy. Their enhanced therapeutic efficiencies when combined with an MF possessing an ultra-low frequency of 20 Hz and medium field strength of 50 mT were verified in the glioma-bearing mice model. Furthermore, we proposed a synergistic therapeutic mechanism. Moreover, the spontaneously magnetized dipoles in Fe_3_O_4_ and unpaired electrons in GA align with the applied external MF, generating a co-directional local additional field that amplifies local MF strength via superposition [22,23]. It precisely enhanced the disruptive effect of the MF on tumor vascular, which hindered the tumor’s nutrient and oxygen supply [24]. This study provides an effective, non-invasive new paradigm for MF-based glioma therapy. It also lays a foundation for the clinical translation of MF-enhancement tumor therapies via magnetic material.

## 2. Materials and Methods

### 2.1. Materials and Instruments

25% (*w*/*w*) Nano Fe_3_O_4_ Dispersion (100–300 nm) was bought from Macklin (Shanghai, China). The gadopentetic acid was purchased from MCE (Shanghai, China). Anhydrous ethanol was bought from Sinopharm Chemical Reagent Co., Ltd. (Shanghai, China). CT-2A mice glioblastoma cells were obtained from BNCC (Beijing, China). PBS, DMEM media, penicillin–streptomycin mixture (PS), trypsin EDTA, environmentally friendly waxing solution, tissue autofluorescence quencher, bovine serum albumin (BSA), DAPI staining reagent and anti-fluorescence quenching mounting medium were obtained from Servicebio (Wuhan, China). Fetal bovine serum (FBS) was acquired from Procell (Wuhan, China).

### 2.2. Cell Culture

All cell lines were cultured in an incubator at 37 °C with an atmosphere of 5.0% CO_2_. CT-2A mice glioma cells were cultured in DMEM supplemented with 1% penicillin–streptomycin solution (PS) and 10% fetal bovine serum (FBS). Cells were passaged at approximately 80% confluency and were detached using trypsin EDTA.

### 2.3. Magnetic Field-Enhancement Therapy Potentiated by Fe_3_O_4_ NPs

Male C57BL/6 mice (3–5 weeks old, 18–22 g) were purchased from Beijing Huafukang Biotechnology Co.,LTD (Beijing, China). A 100 μL suspension of CT-2A cells in PBS (1 × 10^5^ cells per mouse) was subcutaneously injected into the upper thigh of the right hindlimb of each mouse. When the tumor volume reached 100–200 mm^3^, the mice were randomly divided into four groups, ensuring that the initial average tumor volume was consistent across groups (n = 4): (1) control group; (2) MF therapy alone group; (3) Fe_3_O_4_ alone group; (4) Fe_3_O_4_ + MF combination group. On days 1–5 post-grouping, the Fe_3_O_4_ alone and Fe_3_O_4_ + MF groups received intratumoral injections of 50 μ of Fe_3_O_4_ solutions at predetermined concentrations (5 mg/mL, 0.5 mg/mL, 0.05 mg/mL), whereas the control and MF therapy alone groups were injected with an equal volume of PBS. Concomitantly, the MF therapy alone and Fe_3_O_4_ + MF groups were subjected to magnetic field exposure once daily for 4 h from day 1 to day 9 post-grouping. The tumor volume was recorded every day.

### 2.4. Magnetic Field-Enhancement Glioma Therapy Potentiated by GA

The CT-2A tumor-bearing male C57BL/6 mice were also divided into four groups (n = 4): (1) control group; (2) MF therapy alone group; (3) GA alone group; (4) GA + MF combination group. On days 1–5 post-grouping, the GA alone and GA + MF groups received intratumoral injections of GA solution (10 mg/kg, 50 μL, 4 mg/mL), whereas the control and MF therapy alone groups were injected with an equal volume of PBS. Concomitantly, the MF therapy alone and GA + MF groups were subjected to magnetic field exposure once daily for 4 h from day 1 to day 9 post-grouping. A home-made MF generation device was used (CN120919537A, alternating MF, 20 Hz, 50 mT). The tumor volume was recorded every day.

### 2.5. Histopathological Examination

After the treatments finished, the mice were euthanized on day 10. Tumors and major organs were removed and fixed in 4% paraformaldehyde. Following paraffin embedding, tissue sections were prepared and stained with hematoxylin and eosin (H&E). Samples were imaged using a digital pathology slide scanner and analyzed with software.

### 2.6. Mechanism Study

Tumor vasculature was characterized by assessing endothelial cell coverage and microvascular density (MVD) via immunofluorescence staining for CD31, while the cell nuclei was stained with DAPI. Tumor vascular coverage was calculated by measuring the ratio of CD31-positive cells to total cells across 5 randomly selected high-power fields per tumor section. For MVD detection, a single tumor microvessel was defined as a cluster of CD31-positive tumor vascular endothelial cells that was clearly distinguishable from adjacent blood vessels, tumor parenchymal cells, and stromal components. The mean tumor microvascular count across 3 randomly selected high-power fields per tumor section was ultimately defined as the MVD value [25,26]. Immunofluorescence staining was employed to assess the infiltration levels of CD8+ T cells and CD4+ T cells in tumor tissue sections [25,27]. Paraffin sections derived from glioma-bearing mouse models were deparaffinized and the antigen retrieval was performed in EDTA buffer (pH 9.0). Nonspecific binding was blocked with 3% bovine serum albumin (BSA) for 30 min at room temperature. Sections were then incubated overnight at 4 °C with primary antibodies against CD3 (mouse-derived, GB12014, Servicebio, 1:2000), CD4 (rabbit-derived, GB15064, Servicebio, 1:2000), and CD8 (rabbit-derived, GB15068, Servicebio, 1:2000). Then, sections were incubated with HRP-conjugated goat anti-mouse IgG (for mouse-derived CD3+ antibody, GB23301, Servicebio, 1:500) or goat anti-rabbit IgG (for rabbit-derived CD4+/CD8 antibodies, GB23303, Servicebio, 1:500) for 50 min at room temperature. The corresponding TSA was added to incubate in the dark for 10 min: 488 nm for CD3, 555 nm for CD4, and 647 nm for CD8. Nuclei were counterstained with DAPI for 10 min. Fluorescence signals were visualized and acquired using a laser scanning confocal microscope (LSCM). The antibodies used were purchased from Servicebio (Wuhan, China) and Jackson (Shanghai, China).

### 2.7. Statistical Analysis

Graphical representations and statistical analyses were performed using Excel (Office 2021 vesion), GraphPad Prism 10.0, 3DHISTECH (Hungary) CaseViewer2.4 and Image-Pro Plus 6.0 version (Media Cybemetics, USA). Data was statistically analyzed using two-tailed *t*-test or one-way ANOVA. Results were expressed as mean ± SD. The statistical significance was set at *p* > 0.05. N.S. indicate no significance and asterisks indicate significant differences (* *p* < 0.05, ** *p* < 0.01, *** *p* < 0.001, and **** *p* < 0.0001).

## 3. Results

### 3.1. Fe_3_O_4_ NP-Potentiated Magnetic Field Therapy for Glioma

Given the promise of MF therapy in tumor intervention, superparamagnetic Fe_3_O_4_ NPs are ideal for potentiating its therapeutic effects, thanks to their robust magnetic responsiveness.

#### 3.1.1. Tumor Growth Inhibition via Fe_3_O_4_ NP-Potentiated Magnetic Field Therapy

**Figure 1:**
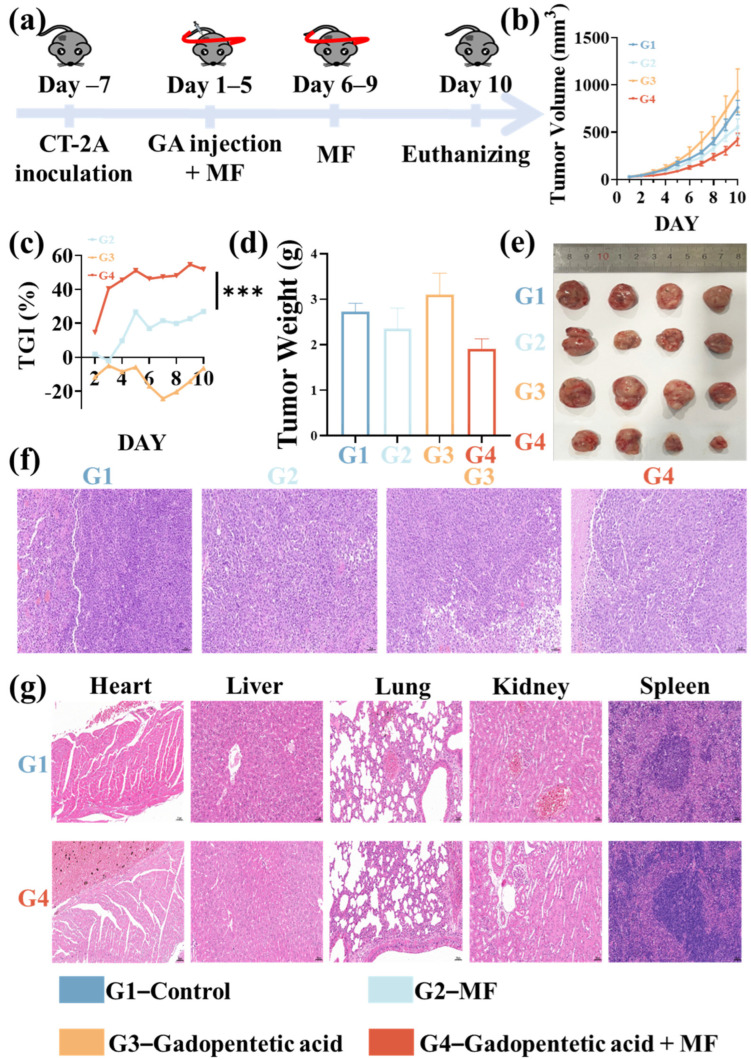
(**a**) Schematic illustration of in vivo treatment schedule. (**b**) Tumor volume dynamics in CT-2A glioma-bearing mice during various treatments. (**c**) Tumor growth inhibition (TGI) rates of different treatments on CT-2A glioma-bearing mice. (**d**) The weight of the dissected tumors at the end of different treatments. (**e**) Photographs of the dissected tumors from the CT-2A glioma-bearing mice after 10 days of different treatments. Representative H&E staining images of (**f**) the tumor tissues and (**g**) major organ sections after different treatments. * *p* < 0.05, ** *p* < 0.01, *** *p* < 0.001, and **** *p* < 0.0001.

To assess the anti-tumor efficacy of Fe_3_O_4_ NPs (5 mg/mL) combined with MF therapy, the tumor volume changes in CT-2A tumor-bearing C57BL/6 mice were monitored (Figure 1a). For comparison, three control groups were also set up: PBS (negative control), MF therapy alone and Fe_3_O_4_ NPs alone. Their tumor volume changes during the treatment duration were recorded (Figure 1b,c). Compared to the PBS control group, all treatment groups exhibited tumor volume reduction, and this effect persisted for the entire 10-day observation period. Especially, the Fe_3_O_4_ NPs and MF combined treatment induced a 64.0% reduction in tumor volume relative to the control group, which was greater than the tumor volume reductions after treatments with Fe_3_O_4_ NPs alone (45.5%) and MF therapy alone (27.0%). It confirmed an enhanced tumor growth inhibition by MF therapy potentiated via Fe_3_O_4_ NPs (Figure 1c). After treatments, the tumor bearing mice were euthanized and the tumors were dissected and weighed. The average tumor weight in the combined treatment group (1.4 ± 0.4 g) was significantly lower than that in the control group (2.5 ± 0.6 g), which was consistent with the tumor volume trend (Figure 1d). It was also lower than the average weights in the MF alone (2.1 ± 0.9 g) and Fe_3_O_4_ NPs alone (1.8 ± 0.5 g) groups, on average. Representative photographs of dissected tumors further confirmed the smallest size of tumors in the combined treatment group (Figure 1e). Moreover, hematoxylin and eosin (H&E) staining of tumor sections revealed extensive and pronounced necrosis in the combined treatment group, indicating effective tumor cell killing (Figure 1f). Notably, no damage to the mice’s major organs was detected, confirming the safety of the MF-enhancement therapy potentiated by Fe_3_O_4_ NPs.

We further validated that the efficiency of the MF-enhancement therapy potentiated by Fe_3_O_4_ NPs is dependent on the concentration of Fe_3_O_4_ NPs. When the concentrations of Fe_3_O_4_ NPs were diluted to 0.5 mg/mL (10-fold dilution) and 0.05 mg/mL (100-fold dilution), the tumor growth inhibition rates dropped to 54.54% and 35.77%, respectively (Supporting information, Figures S1 and S2). These results emphasized that Fe_3_O_4_ NP concentration was a key determinant of the synergistic interaction between Fe_3_O_4_ NPs and MF therapy, with the concentration of 5 mg/mL being optimal for maximizing therapeutic efficacy.

To elucidate the mechanism underlying the optimal Fe_3_O_4_ NP (5 mg/mL)-enhanced MF therapy, the effect of this combined intervention on key components of the tumor microenvironment (TME)—specifically tumor neovascularization and immune cell infiltration—was quantitatively assessed.

#### 3.1.2. Tumor Neovascularization via Fe_3_O_4_ NP-Potentiated Magnetic Field Therapy

**Figure 2:**
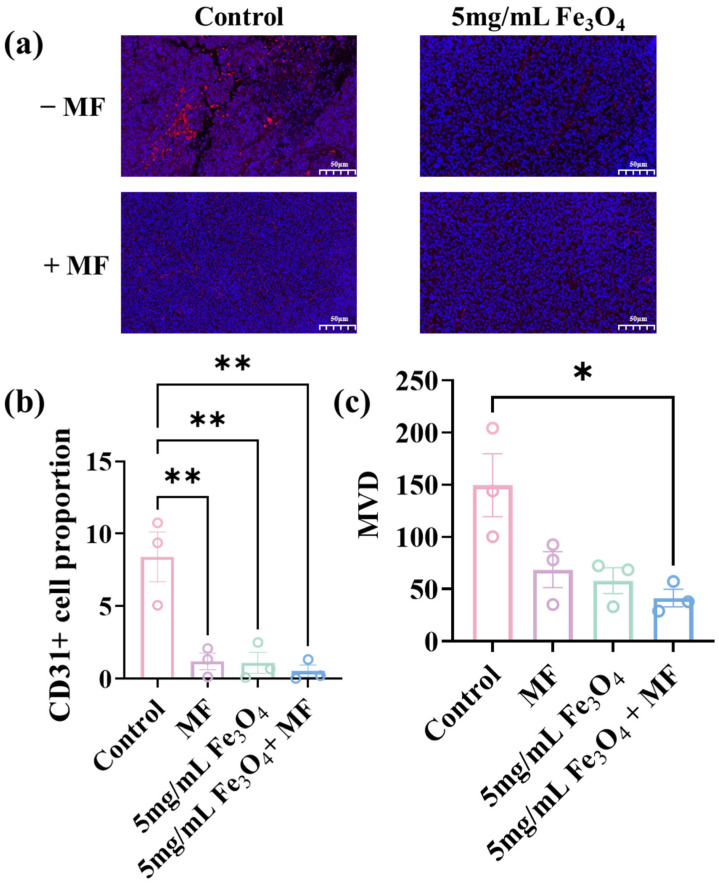
(**a**) Immunofluorescence staining of endothelial cells (anti-CD31 antibody, red; DAPI stained cell nucleus, blue). (**b**) Quantitative analysis of CD31 fluorescence intensity. (**c**) MVD values following different treatments. The scale bar is 50 μm. * *p* < 0.05, ** *p* < 0.01, *** *p* < 0.001, and **** *p* < 0.0001.

To assess tumor neovascularization, CD31+ endothelial cells—a well-established marker of neovascularization—were analyzed in the tumor tissues after different treatments (Figure 2a). The intratumoral CD31-positive cell percentage in the Fe_3_O_4_ NPs + MF combined treatment group was reduced the most dramatically on average, reaching about half of that in the control group (Figure 2b). It indicated a significant loss of vascular endothelial cell mass. The average microvessel density (MVD) values for the standalone therapy groups were 58.1 after Fe_3_O_4_ NPs treatment alone and 68.7 after MF therapy alone, respectively (Figure 2c). It showed a significant decline on average compared to that in the control group (149.6). The average MVD showed the largest decrease to 41.4 in the Fe_3_O_4_-enhanced MF therapy group, which was the only group that showed a statistical difference from the control group.

At the mechanistic level, Fe_3_O_4_ NPs have been reported to generate localized mechanical stress or mild hyperthermia upon MF exposure [28,29,30]. These effects synergistically exacerbated endothelial cell apoptosis and basement membrane degradation, thereby amplifying vascular disruption compared to MF therapy alone. In this study, we also proposed that superparamagnetic Fe_3_O_4_ could act as a magnetic field amplifier and be combined with the iron metabolism-targeted intervention, which could amplify the MF effect [31,32]. This amplification effect was positively correlated with the concentration of Fe_3_O_4_ NPs. The results confirmed that Fe_3_O_4_ NPs’ concentration-dependent MF effect amplification was indispensable for enhancing MF-induced tumor vascular damage, thereby potentiating the anti-angiogenic and tumor-suppressive effects of the combined therapy. This vascular-targeting mechanism ultimately restricted nutrient and oxygen delivery to proliferating tumor cells, contributing to the observed tumor growth inhibition [33,34,35].

#### 3.1.3. Immune Cells Infiltration via Fe_3_O_4_ NP-Potentiated Magnetic Field Therapy

**Figure 3:**
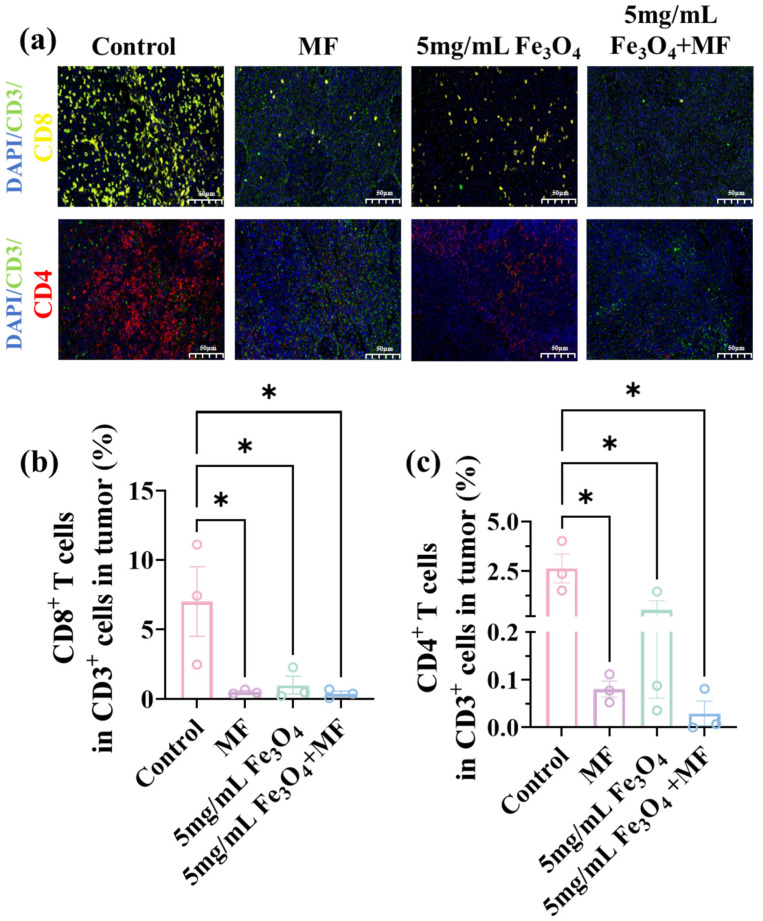
(**a**) Immunofluorescence staining images of CD3+CD4+ helper T cells and CD3+CD8+ cytotoxic T lymphocytes (CD3+ cells: green; CD8+ cells: yellow; CD4+ cells: red; DAPI stained cell nucleus, blue). Quantitative proportion analysis of (**b**) CD8+ T cells and (**c**) CD4+ T cells among total CD3+ cells following different treatments. The scale bar is 50 μm. * *p* < 0.05.

Tumor vascular dysfunction was a well-recognized barrier to effective cancer immunotherapy, as disorganized or hypoperfused vasculature limited the delivery of immune effector cells into the tumor parenchyma [25,36]. To investigate whether MF-enhancement therapy via superparamagnetic Fe_3_O_4_ with optimal concentration of 5 mg/mL modulated the tumor immune microenvironment, the infiltration of key immune effectors, CD3+CD4+ helper T cells and CD3+CD8+ cytotoxic T lymphocytes (CTLs), were quantified using immunofluorescence staining (Figure 3a). Representative images revealed sparse infiltration of CD3+CD4+ and CD3+CD8+ T cells in tumors after all the treatments compared to the control group, with the most pronounced reduction observed in the Fe_3_O_4_-enhanced MF therapy group. In contrast, tumors in the control group exhibited dense, widespread infiltration of these T cell subsets (Figure 3a). This observation was further substantiated by quantitative analysis (Figure 3b). The average proportion of CD8+ T cells among total CD3+ cells in the MF-enhancement therapy group was only 0.4%, representing a ~94.3% reduction compared to the control group (7.0%) and a ~60.0% reduction compared to the standalone Fe_3_O_4_ NPs group (1.0%). It was also ~20.0% lower than that in the standalone MF therapy group (0.5%) (Figure 3b). Similarly, the average infiltration proportion of CD3+CD4+ T cells in the MF-enhancement therapy group was 0.03%, which was ~99.0% lower than the control group (2.63%), ~94.5% lower than the standalone Fe_3_O_4_ NPs group (0.53%) and ~62.5% lower than the standalone MF therapy group (0.08%).

This reduction in T cell infiltration aligned with the vascular disruption data. Specifically, impaired angiogenesis, those characterized by reduced CD31+ vessels and MVD, likely physically blocked T cell extravasation from the circulation into tumors, while hypoxic conditions secondary to vascular rarefaction may further dampen immune cell recruitment [25,37]. Collectively, the diminished infiltration of CD4+ and CD8+ T cells was a transient secondary effect caused by the effective vascular disruption by Fe_3_O_4_ NP-mediated MF-enhancement therapy, reinforcing that its anti-angiogenic activity was a key driver of the observed anti-tumor effects.

### 3.2. GA-Potentiated Magnetic Field Therapy for Glioma

Building on the proposed magnetic field (MF) effect amplification mechanism via superparamagnetic Fe_3_O_4_, which was distinct from the heat generation of conventional magnetic hyperthermia or the mechanical stress of magnetomechanical therapy, we further sought to identify another candidate MF amplifier. Gadopentetic acid (GA) was chosen as the second candidate due to its intrinsic paramagnetic property derived from Gd^3+^ ions with seven unpaired electrons, which could align with the applied MF to mediate MF effect amplification. It was consistent with our proposed mechanism while lacking the heat-generating or stress-inducing effects of traditional magnetic therapies. Its distinct material nature enabled cross-validation to rule out material-specific artifacts and confirmed the generalizability of the MF effect amplification mechanism.

#### 3.2.1. Tumor Growth Inhibition via GA-Potentiated Magnetic Field Therapy

**Figure 4:**
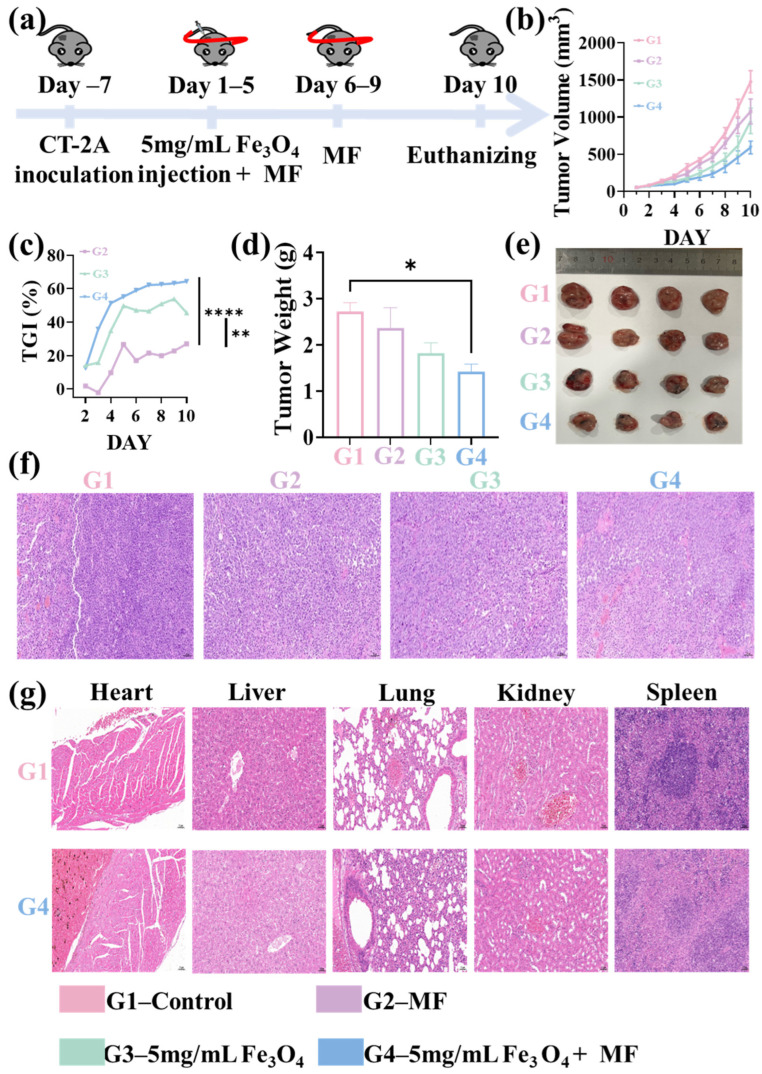
(**a**) Schematic illustration of the treatment schedule. (**b**) Effects of various treatments on CT-2A glioma growth dynamics in vivo. (**c**) Effects of different treatments on TGI rates in CT-2A glioma-bearing mice. (**d**) Dissected tumor weight measured at the end of the treatment regimen. (**e**) Representative photographs of excised tumors from CT-2A glioma-bearing mice in different groups at the post-treatment endpoint on the 10th day. Representative H&E staining images of (**f**) the tumor tissues and (**g**) major organ sections from the different treatment groups. * *p* < 0.05, ** *p* < 0.01, *** *p* < 0.001, and **** *p* < 0.0001.

To assess the in vivo therapeutic efficacy of MF combined with GA, CT-2A glioma-bearing C57BL/6 mice were employed as the animal model (Figure 4a). Over the initial 5-day treatment period, mice in the MF + GA combination group underwent daily intratumoral injections of GA (10 mg/kg, dissolved in 50 μL of sterile saline). At the same time, these mice were exposed to the MF for 4 h per day, with tumor volumes measured once every day. It could be seen that GA alone failed to inhibit tumor growth, leading to larger tumor volumes than those in the control group (Figure 4b–e). In comparison, the MF + GA group showed an obvious tumor volume reduction, with the maximum tumor inhibition rate of 55.0%, the efficiency of which was significantly greater than that of MF therapy alone (28.0%) (Figure 4b,c).

After the treatments, mice were euthanized and tumors were excised for weight measurement and taking photos. Tumor weights after different treatments were consistent with their tumor volume. The MF + GA group exhibited the lowest mean tumor weight (1.5 ± 0.5 g) (Figure 4d). Representative tumor images further confirmed the enhanced therapeutic effect of GA-potentiated MF therapy (Figure 4e). H&E staining results revealed that tumors treated with GA-potentiated MF therapy displayed more extensive necrotic regions compared to untreated tumors or those treated with MF therapy alone (Figure 4f). Moreover, histological examination of major organs (heart, liver, spleen, lung, kidney) in mice after GA-potentiated MF therapy showed no obvious pathological damage, illustrating its good biocompatibility. Collectively, these findings validated the functional role of GA in amplifying MF-induced tumor cytotoxicity, supporting its potential as a synergistic agent for MF-based cancer therapy.

#### 3.2.2. Tumor Neovascularization via GA-Potentiated Magnetic Field Therapy

**Figure 5:**
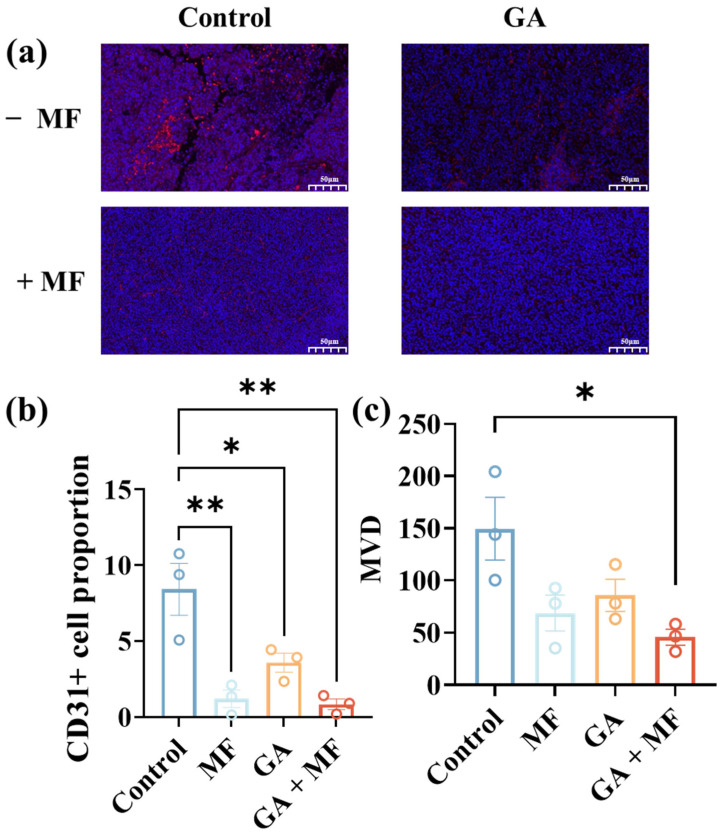
(**a**) Immunofluorescence staining for CD31-positive tumor vascular (CD31 positive cells: red fluorescence; DAPI stained cell nucleus: blue). (**b**) CD31-positive cell proportion within the tumor sections after different treatments. (**c**) Effects of different treatments on MVD in tumor sections. The scale bar is 50 μm. * *p* < 0.05, ** *p* < 0.01, *** *p* < 0.001, and **** *p* < 0.0001.

Similar to Fe_3_O_4_, GA-potentiated MF therapy also showed an inhibitory effect on tumor vascular. Immunofluorescence staining images for CD31 revealed a significant reduction in fluorescence intensity within tumor tissues following GA-potentiated MF treatment (Figure 5a). Specifically, the average CD31+ cell proportion in the tumors in the GA + MF combined therapy group was reduced to 0.8%, which was a substantially greater than the control group (8.4%), GA therapy alone group (3.6%), and MF therapy alone group (1.2%) (Figure 5b). It directly supported tumor vascular targeting by the MF therapy, with the effect enhanced by GA. Similar to the Fe_3_O_4_ NPs, only the MVD in the GA + MF combination therapy group (45.7) showed a statistical difference from the control group (149.6). The average MVD value after the GA treatment alone (85.7) and MF treatment alone (68.7) were also reduced on average compared to the control group, but at a lower level (Figure 5c).

It demonstrated that GA-potentiated MF therapy effectively disrupted established tumor vascular networks and suppressed tumor neovascularization. It further supported that GA synergized with MF targeted tumor angiogenesis, which was critical for tumor progression, thereby enhancing therapeutic efficacy.

#### 3.2.3. Immune Cells Infiltration via GA-Potentiated Magnetic Field Therapy

**Figure 6:**
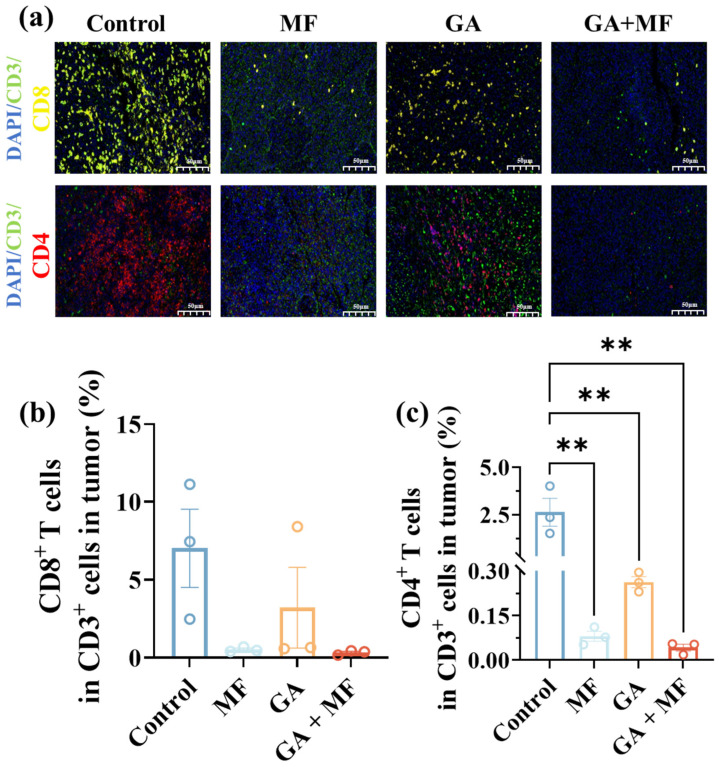
(**a**) Immunofluorescence staining for CD3+CD4+ T cells and CD3+CD8+ T cells (CD3+ cells: green; CD8+ cells: yellow; CD4+ cells: red; DAPI stained cell nucleus, blue). Quantitative proportion analysis of (**b**) CD8+ T cells and (**c**) CD4+ T cells among total CD3+ cells following different treatments. The scale bar is 50 μm. * *p* < 0.05, ** *p* < 0.01, *** *p* < 0.001, and **** *p* < 0.0001.

Parallel assessment of immune cell infiltration further corroborated the anti-angiogenic efficacy of GA-potentiated MF therapy. Immunofluorescence staining for CD3+CD4+ and CD3+CD8+ T cells, in conjunction with quantitative image analysis, revealed striking differences in intratumoral T cell densities between the control group and all treatment groups (GA monotherapy, MF monotherapy, and GA + MF combined therapy) (Figure 6a–c). Specifically, compared to the control group, the GA + MF combined therapy group exhibited a significant reduction in intratumoral CD3+CD4+ T cell percentage by 98.0% and in CD3+CD8+ T cell percentage by 95.0%. It was also substantially reduced compared to those in the GA monotherapy group (CD4+ T cells reduced by 80.8%; CD3+CD8+ T cells reduced by 89.7%) and MF monotherapy group (CD3+CD4+ T cells reduced by 37.5%; CD3+CD8+ T cells reduced by 37.7%).

This attenuation of CD3+CD4+ and CD3+CD8+ T cell infiltration into the tumor reflected a consequence of tumor vascular disruption. This observation was consistent with the previous report, that dysfunctional tumor vasculature hindered effective immune cell infiltration into the TME [25]. It further reinforced the conclusion that GA-potentiated MF therapy effectively compromised tumor angiogenesis by amplifying local MF effects.

## 4. Discussion

This study demonstrated that both superparamagnetic Fe_3_O_4_ NPs and paramagnetic GA significantly potentiated the anti-tumor efficacy of an externally applied magnetic field in a glioma model, primarily through the suppression of tumor angiogenesis. Specifically, Fe_3_O_4_ NP-potentiated MF therapy and GA-potentiated MF therapy achieved TGI rates of ~64% and ~55%, respectively, markedly exceeding the ~27% TGI rate observed with MF therapy alone. This enhanced efficacy was not merely additive but rather reflective of a synergistic interaction, wherein these MF amplifiers augmented local MF intensity within the TME. The difference in their enhancement potency was proposed to stem from inherent differences in their magnetic field effect amplification capabilities, which arose from fundamental distinctions in magnetic properties and magnetization mechanisms between the two magnetic materials [38].

Paramagnetic materials (e.g., GA with seven unpaired electrons) derived magnetization from the oriented alignment of intrinsic atomic/molecular magnetic moments [39,40]. Under an external MF, a subset of these magnetic moments aligned directionally with MF, generating a magnetization intensity parallel to the external MF. However, individual atomic/molecular magnetic moments in paramagnetic materials were extremely weak [41,42,43]. It resulted in low overall magnetization, resulting in a modest MF effect amplification. In contrast, superparamagnetic materials (e.g., Fe_3_O_4_ NPs) were nanoscale ferromagnetic/ferrimagnetic particles with a magnetization mechanism originating from the directional alignment of the total magnetic moments of single-domain particles. Under an external MF, these high-magnetic-moment single-domain particles aligned efficiently and directionally with MF, producing a strong magnetization, thus enabling significant amplification of the local MF [44,45].

In this work, Fe_3_O_4_ NPs and GA were applied as MF amplifiers for tumor therapy, which was different from their conventional roles as hyperthermia mediators or MRI contrast agents. It enhanced the effect of MF on the disruption of tumor vasculature, which was evidenced by significant reductions in CD31 expression and MVD. This vascular disruption further contributed to reduced intratumoral infiltration of CD4+ and CD8+ T cells [25]. The consistent anti-angiogenic and immune infiltration-suppressive effects across two chemically distinct amplifiers (Fe_3_O_4_ NPs and GA) supported a shared mechanism, i.e., local MF effect amplification, rather than compound-specific biochemical effects. This cross-validation strengthened the robustness of our proposed mechanism and highlighted the broader potential of MF effect amplification as a generalizable strategy to enhance MF-based cancer therapy.

## 5. Conclusions

In conclusion, our findings established a novel strategy for long-acting, safe, magnetically mediated precision oncology for glioma treatment. Distinguishing from the traditional reliance of magnetic field therapy on high frequency and high field strength, the non-thermal physical effects and biological regulatory effects of a magnetic field with an ultra-low frequency of 20 Hz and medium field strength of 50 mT were used as the core therapeutic driving force. By repurposing conventional magnetic functional agents, i.e., Fe_3_O_4_ NPs and GA, to amplify the local magnetic field effect, effective suppression of tumor angiogenesis and subsequent tumor growth were achieved. On the one hand, GA broke through the traditional limitation of contrast agents that only provided imaging but no treatment function. On the other hand, Fe_3_O_4_ focused on non-thermal magnetic response, overturning its classic application in magnetic therapy as only a magnetic hyperthermia medium. What they have in common is that they could both generate a co-directional local additional field that amplified local MF strength, which was generated through the alignment of spontaneously magnetized dipoles in Fe_3_O_4_ and unpaired electrons in GA with the applied external MF. It precisely enhanced the anti-tumor effect of MF. In the glioma-bearing mice model, this strategy reduced tumor volume significantly, increasing the tumor growth inhibition (TGI) rate from 27.0% (MF therapy alone) to 64.0% (MF combined with Fe_3_O_4_ NPs) and 55.0% (MF combined with GA). This strategy addressed a critical limitation of standalone MF therapy and opened a new avenue for enhancing the therapeutic efficacy of physical field-mediated cancer therapies. At this stage, the effective tumor inhibition was attributed to the reduction in tumor vasculature, which blocked the tumor’s nutrient supply, while it resulted in the decrease in T cell infiltration. In the long run, vascular normalization regulation and immune activation could be combined with this strategy to achieve a balance of effective tumor inhibition, moderate vascular preservation and the promotion of immune cell infiltration to decrease the risk of tumor recurrence/metastasis.

## Data Availability

The original contributions presented in this study are included in the article/Supplementary Material. Further inquiries can be directed to the corresponding authors.

## Supplementary Materials

The following supporting information can be downloaded at: https://www.mdpi.com/article/10.3390/pharmaceutics18030319/s1, Figure S1: In vivo therapeutic effect of 10-fold diluted Fe_3_O_4_ NPs suspension (0.5 mg/mL) in combination with magnetic field; Figure S2: In vivo therapeutic effect of 100-fold diluted Fe_3_O_4_ NPs suspension (0.05 mg/mL) in combination with magnetic field.

## Abbreviations

The following abbreviations are used in this manuscript:

GA	Gadopentetic acid
MF	Magnetic field
TGI	Tumor growth inhibition
MVD	Microvessel density
TME	Tumor microenvironment
CTLs	Cytotoxic T lymphocytes
