# Supplementary material for: Repurposing Conventional Magnetic Functional Agents: A Novel Strategy for Long-Acting, Safe, Magnetically Mediated Precision Oncology
Source: Pharmaceutics. 2026 Mar 2;18(3):319. doi: 10.3390/pharmaceutics18030319 (PMC13028909; doi:10.3390/pharmaceutics18030319)
Supplement: Supplementary file 1 [file pharmaceutics-18-00319-s001.zip › pharmaceutics-4079787-supplementary.pdf]

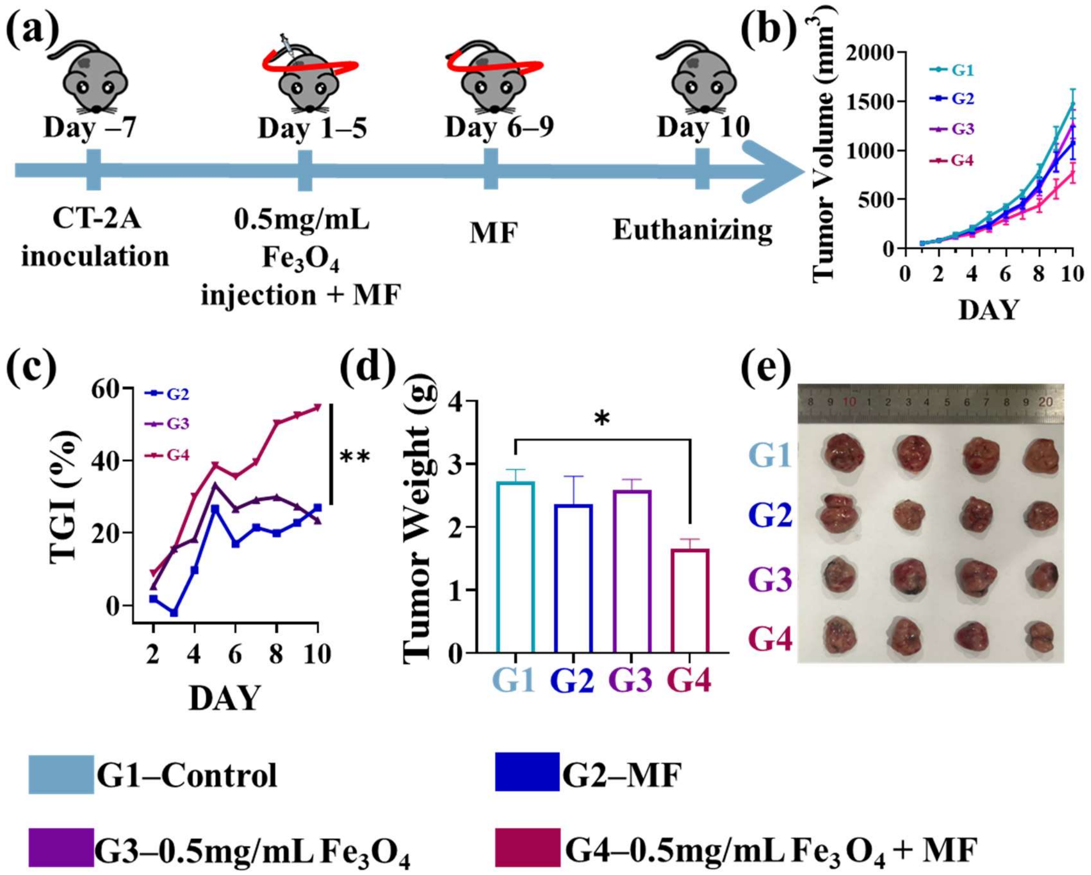


**Figure S1** In vivo therapeutic effect of 10-fold diluted 0.5mg/mL of $Fe_3O_4$ NPs suspension in combination with magnetic field.

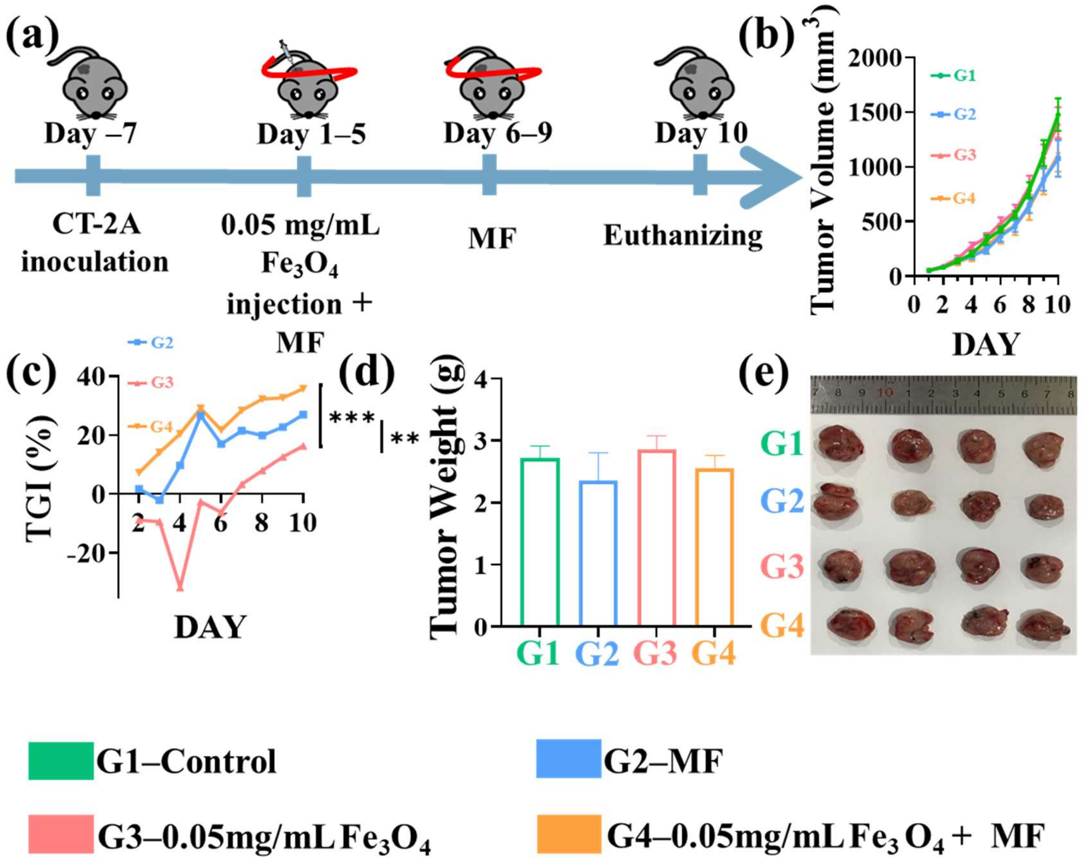


**Figure S2** In vivo therapeutic effect of 100-fold diluted 0.05mg/mL of $Fe_3O_4$ NPs suspension in combination with magnetic field.
